# Targetless LiDAR–Camera Extrinsic Calibration via Class-Agnostic Boundary Mask Alignment and SPSA-Based Optimization

**DOI:** 10.3390/s26051501

**Published:** 2026-02-27

**Authors:** Han-You Jeong, Woo-Hyuk Son, Dong-Wook Shin, Kyuna Cho, Minwoo Chee, Tae (Tom) Oh

**Affiliations:** 1 Department of Electrical Engineering, Pusan National University, Busan 46241, Republic of Korea; rtson24@naver.com (W.-H.S.); b5150624@naver.com (K.C.); michael1998@naver.com (M.C.); 2 Department of Computer Science and Engineering, Pusan National University, Busan 46241, Republic of Korea; dntks1942@pusan.ac.kr; 3 School of Information, Rochester Institute of Technology (RIT), Rochester, NY 14623, USA; thoics@rit.edu

**Keywords:** targetless sensor calibration, LiDAR–camera extrinsic calibration, image-plane projection, class-agonistic segmentation, simultaneous perturbation stochastic approximation (SPSA)

## Abstract

Targetless LiDAR–camera extrinsic calibration remains challenging due to unreliable cross-modal correspondences and sensitivity to initialization. We present a targetless extrinsic calibration framework based on class-agnostic boundary mask alignment in a shared image-plane representation. This scheme first constructs consistent LiDAR–camera mask pairs from image-plane depth and intensity projections of LiDAR data and camera images. It then obtains robust initial pose candidates through bounded rotation-only global initialization and refines them using a computationally efficient stochastic gradient approximation to estimate the optimal extrinsic parameters. Experiments on the KITTI benchmark demonstrate a superior accuracy–runtime trade-off compared with a segmentation-based global optimization baseline, while real-world driving tests confirm stable cross-modal alignment under vibration and inter-modal timing jitter.

## 1. Introduction

The advancement of autonomous driving technologies critically depends on multi-sensor perception systems, among which the fusion of LiDAR and cameras has become a cornerstone for achieving high-precision object recognition and spatial understanding. A prerequisite for effective fusion is the accurate alignment of their coordinate systems, commonly referred to as extrinsic calibration. Calibration errors can directly propagate into perception pipelines, leading to mislocalized objects and inaccurate distance estimation and ultimately degrading perception accuracy and driving safety [1,2].

Conventional LiDAR–camera calibration approaches predominantly rely on physical targets such as checkerboards or custom calibration boards, where corresponding points are extracted and projection errors are minimized [3,4,5]. These approaches can achieve high accuracy in controlled laboratory conditions but require precise installation and constrained capture setups, which make them impractical for large-scale or outdoor driving environments. To overcome these limitations, targetless calibration approaches have been actively explored. These approaches leverage sensor data directly, aligning structural features such as edges, contours, or projection regions across modalities, and in some cases integrating them into deep-learning pipelines [6,7]. Although more flexible, targetless approaches often depend on specific environmental structures or task-driven learning, and their performance is typically sensitive to initialization. In practice, this sensitivity is further amplified by imperfect cross-modal associations and real-world disturbances (e.g., sensor vibration and inter-modal timing jitter). Moreover, exhaustive exploration of the six degree of freedom (DoF) space can be computationally demanding. This motivates a targetless calibration framework that builds reliable cross-modal associations and refines extrinsics efficiently from a coarse but informative initialization, enabling accurate and practical deployment across diverse driving scenarios.

Building on these observations, we propose PAIRS-Calib (Pairwise Assignment and Iterative Refinement via SPSA), a targetless LiDAR–camera extrinsic calibration framework that estimates 6-DoF extrinsics from class-agnostic boundary mask (BM) alignment in a common image-plane representation. Using class-agnostic segmentation, BMs are extracted from both the projected LiDAR maps and the RGB image. PAIRS-Calib leverages image-plane depth and intensity projections of LiDAR data to enable direct cross-modal comparison via overlap- and boundary-level consistency, without requiring explicit 2D–3D point correspondences. Our approach first forms consistent projected LiDAR–camera mask pairs by jointly considering image-plane overlap/coverage and a lightweight shape-consistency cue. It then computes coarse pose initialization candidates cost-efficiently through bounded rotation-only global exploration, and refines them using a computationally efficient Simultaneous Perturbation Stochastic Approximation (SPSA). Both coarse initialization and refinement exploit effective geometric objectives based on boundary-to-boundary distances (via a distance transform) and intersection-over-union (IoU) consistency between projected and image-derived masks. Unlike motion-cue-based schemes that rely on sensor odometry for miscalibration detection and correction [8], PAIRS-Calib performs calibration from boundary alignments, enabling adaptive recalibration without motion estimation.

The main contributions of this paper are three-fold: (1) PAIRS-Calib, a targetless image-plane calibration framework with overlap- and shape-aware mask pairing; (2) a cost-efficient coarse initialization and SPSA-based refinement pipeline using boundary-distance and IoU objectives for 6-DoF recovery; and (3) extensive validation on a public benchmark and a real driving platform, demonstrating a favorable accuracy–runtime trade-off and stable performance under practical disturbances.

The remainder of this paper is organized as follows: Section 2 reviews related work and positions PAIRS-Calib within targetless LiDAR–camera calibration. Section 3 defines the problem formulation, and then Section 4 presents the proposed PAIRS-Calib framework. Section 5 evaluates its accuracy, efficiency, and practical applicability, and finally, Section 6 concludes this paper.

## 2. Related Work

Targetless LiDAR–camera calibration has been explored under diverse representations and inference strategies [1,2]. To clarify the design space concisely, Table 1 summarizes representative approaches along four axes: LiDAR representation, camera representation, matching space (3D–2D vs. 2D–2D), and inference paradigm. This section follows the same taxonomy to systematically compare representation and inference strategies and to position class-agnostic approaches within this design space.

Beyond the division between target-based and targetless strategies, LiDAR–camera calibration approaches can be classified by LiDAR data representation. Early studies relied on raw point clouds aligned with 2D image features [3,4,11], but establishing reliable 2D–3D correspondences has remained challenging [1,2]. To address this gap, Bird’s-Eye View (BEV) projections have been widely adopted, offering global geometric layouts and improved robustness in large-scale scenes [9,12], though often at the expense of fine detail due to sparse sampling. As an alternative, Front-View (FV) projections preserve the native perspective of sensors, enable direct point-to-pixel correspondence, and facilitate boundary-level alignment [13,14,15]. Although FV alignment reduces calibration to a 2D–2D problem with limited depth cues, it provides a natural basis for extracting geometry-consistent features. In this work, we adopt FV as a practical image-plane projection domain and focus on boundary-level alignment that is applicable to general image-plane representations.

From the perspective of camera representation, targetless approaches differ in what image-domain cues are extracted for cross-modal consistency. Classical pipelines often rely on handcrafted edges or gradients [11,16], while instance/semantic segmentation introduces mask-level structures but may be constrained by category priors and domain specificity [17]. Recent progress in class-agnostic segmentation enables extracting object boundaries without semantic labels [18], offering a more general way to obtain mask/boundary cues that can be compared against projected LiDAR structures. This trend is reflected in Table 1, where camera-side cues range from edge detection [6] to class-agnostic masks [10] and learned convolutional neural network (CNN) features [7].

With a chosen representation, targetless calibration can be organized by matching space: 3D–2D alignment that couples 3D LiDAR structures with 2D image cues, and 2D–2D alignment that compares projected LiDAR representations with image-domain features on a common plane [2]. 3D–2D formulations can leverage explicit geometry but often require careful correspondence design and can be sensitive to sparsity and occlusion [3,11]. In contrast, 2D–2D formulations reduce correspondence burden by operating in a common projection space, but their success hinges on the stability of the projection and the reliability of extracted cues. Table 1 illustrates this split: MFCalib and CalibAnything operate in 3D–2D matching [6,10], whereas BEVCalib and CalibRefine adopt 2D–2D matching in BEV or FV space [7,9].

Given the representation and matching space, approaches differ most clearly in their inference paradigm. Geometric optimization aligns explicit edge/mask structures through handcrafted objectives (e.g., MFCalib) [6]. Global optimization seeks extrinsics that maximize projection consistency, often at higher computational cost (e.g., CalibAnything) [10]. Learning-based pipelines align latent representations and perform refinement through trained networks (e.g., BEVCalib and CalibRefine) [7,9]. Overall, these paradigms highlight a recurring tension between robustness to imperfect observations and practical computational cost, which is a central concern in real-world targetless calibration [1,2].

In this context, CalibAnything demonstrates the attractiveness of class-agnostic cues for targetless calibration by leveraging Segment Anything Model (SAM)-derived masks [10,18]. By casting calibration as a projection-consistency optimization over segmentation masks, it removes reliance on predefined object categories and handcrafted correspondences, thereby broadening applicability across diverse environments. However, its global optimization formulation introduces two practical challenges. First, segmentation uncertainty may yield unreliable or partially inconsistent mask instances under occlusion or sparse observations, affecting the stability of a single global objective. Second, the computational burden of global search can increase when robustness over wide pose perturbations is required. These observations suggest that while class-agnostic masks provide a powerful representation, their effectiveness depends critically on how uncertainty and optimization are structured.

Motivated by these observations, PAIRS-Calib is positioned as a class-agnostic, image-plane BM calibration framework that retains the generality of class-agnostic cues while explicitly addressing segmentation uncertainty and optimization sensitivity. Rather than relying on a single global optimization trajectory, PAIRS-Calib organizes estimation around multiple pairwise pose hypotheses derived from projected LiDAR–camera mask pairs. Concretely, it first constructs consistent matching pairs in the image plane, then computes cost-efficient coarse initialization candidates through bounded rotation-only exploration and finally performs iterative refinement using SPSA [19]. By separating pair construction, coarse exploration, and lightweight stochastic refinement, this framework reduces dependence on any single mask instance and mitigates the risk of local-minimum entrapment. By narrowing the search space through bounded coarse initialization and refining candidates via SPSA, the proposed framework achieves a practical accuracy–runtime balance under real-world disturbances, offering improved robustness without relying on large-scale global exploration.

## 3. Formulation of the LiDAR–Camera Extrinsic Calibration Problem

**Figure 1:**
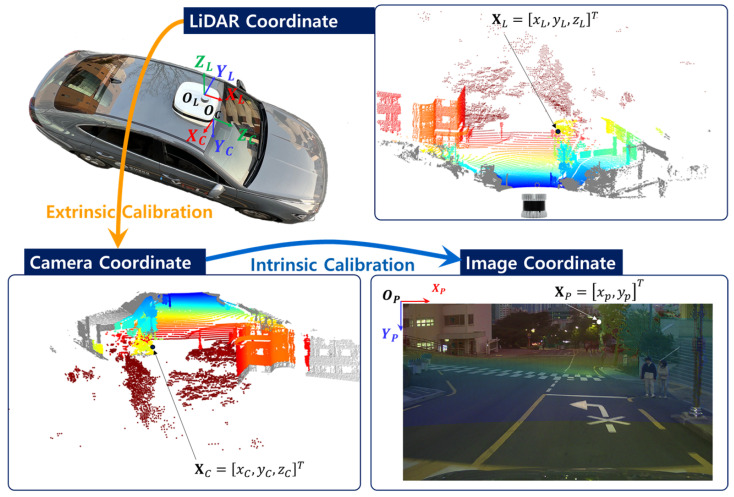
Coordinate transformation from the LiDAR coordinate system to the image coordinate system in the extrinsic calibration framework. The 3D point cloud is first expressed in the LiDAR coordinate frame and transformed into the camera coordinate frame via extrinsic calibration, followed by projection onto the image plane through intrinsic calibration. The colors of all coordinate systems represent depth values, visualized using a colormap.

LiDAR–camera calibration broadly refers to estimating the geometric relationship between LiDAR and camera sensors so that their measurements can be expressed in a common coordinate framework [1,2]. In the case of extrinsic calibration, this typically involves recovering the 6-DoF rigid transformation between the LiDAR and camera coordinate systems. We focus on a targetless extrinsic calibration scenario that operates directly on sensor data collected during driving, without the need for artificial calibration targets or controlled setup procedures. In this work, this targetless formulation is addressed by estimating the extrinsic parameters through structural consistency between projected LiDAR measurements and image-domain features in a shared image-plane representation. As shown in Figure 1, three coordinate systems are considered: the LiDAR coordinate system with origin $O_{L}$ and points $X_{L}={\left[x_{L},y_{L},z_{L}\right]}^{T}$ expressed in axes $\left(X_{L},Y_{L},Z_{L}\right)$, the camera coordinate system with origin $O_{C}$ and points $X_{C}={\left[x_{C},y_{C},z_{C}\right]}^{T}$ expressed in axes $\left(X_{C},Y_{C},Z_{C}\right)$, and the image coordinate system with origin $O_{P}$ and points $X_{P}={\left[x_{P},y_{P}\right]}^{T}$ defined in axes $\left(X_{P},Y_{P}\right)$. The transformation from the LiDAR frame to the camera frame is determined by the extrinsic parameters, while the projection onto the image plane is governed by the intrinsic parameters of the camera.

The extrinsic parameters, denoted by the vector θ, describe the relative pose between the LiDAR and the camera coordinate systems:

(1) $$\theta ={\left[\Delta \phi ,\Delta \theta ,\Delta \psi ,\Delta x,\Delta y,\Delta z\right]}^{T},$$

where the first three elements (Δϕ,Δθ,Δψ) represent roll, pitch, and yaw rotations, respectively, and the last three (Δx,Δy,Δz) denote the translation vector $t\in R^{3}$, with its components corresponding to the *x*, *y*, and *z* axes, respectively. The rotation matrix R∈SO(3) is constructed as a composition of elemental rotations in the *Z*–*Y*–*X* order:

(2) $$R=R_{z}\left(\Delta \psi \right)\;\cdot \;R_{y}\left(\Delta \theta \right)\;\cdot \;R_{x}\left(\Delta \phi \right).$$

The rigid-body transformation from LiDAR to camera coordinates is written in homogeneous form as

(3) $$T_{LC}=\left[\begin{array}{cc} R & t\\ 0_{1\times 3} & 1 \end{array}\right]\in R^{4\times 4}.$$

Given a LiDAR point $X_{L}$, its homogeneous representation $X_{L}^{h}={\left[x_{L},y_{L},z_{L},1\right]}^{T}$ is mapped to the corresponding point $X_{C}^{h}$ in the camera frame:

(4) $$X_{C}^{h}=T_{LC}\;\cdot \;X_{L}^{h}.$$

The projection of $X_{C}$ onto the image coordinates $X_{P}={\left[x_{P},y_{P}\right]}^{T}$ is then obtained using the intrinsic matrix $K\in R^{3\times 3}$:

(5) $$X_{P}^{h}=\left[\begin{array}{c} x_{P}\\ y_{P}\\ 1 \end{array}\right]=\frac{1}{z_{C}}K\;\cdot \;X_{C}.$$

Finally, the calibration task can be formulated as the following optimization problem:

(6) $$\theta^{*}=\arg\min_{\theta }\;L\left(\theta \right),$$

where L(θ) denotes a geometric misalignment loss that measures the discrepancy between projected LiDAR structures and image-derived structural cues in the image plane.

In summary, the problem of LiDAR–camera extrinsic calibration is to estimate θ such that the projected LiDAR points $X_{P}$ achieve maximum consistency with image features. The specific choice of distance metric L(·) and the optimization strategy are detailed in the next section presenting the PAIRS-Calib framework.

## 4. The Proposed PAIRS-Calib Framework

**Figure 2:**
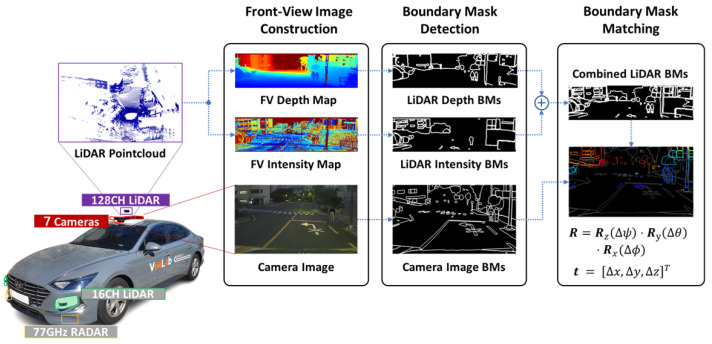
System overview of the proposed PAIRS-Calib framework: LiDAR pointclouds are projected into FV depth and intensity maps, and the RGB camera image is used as a visual counterpart. Class-agnostic BMs are extracted from each modality to capture structural outlines. The calibration is then performed by constructing reliable projected LiDAR–camera BM pairs, generating cost-efficient coarse initialization candidates, and refining extrinsics via SPSA.

This section presents a detailed description of the proposed PAIRS-Calib framework. This framework is designed to address targetless LiDAR–camera calibration under real-world conditions, where irregular sampling density, viewpoint differences, and segmentation noise make direct 2D–3D correspondence unreliable. The overall system configuration is illustrated in Figure 2.

To ensure clarity and modularity, this framework is structured hierarchically into input construction, procedural overview, and stage-wise optimization components. Section 4.1 describes input data generation via image-plane projection and BM construction; Section 4.2 provides an overview of the overall PAIRS-Calib procedure; and Section 4.3, Section 4.4 and Section 4.5 detail each stage of PAIRS-Calib procedure. The central idea of PAIRS-Calib is to derive boundary representations consistently from both LiDAR and camera modalities and to use these representations as reliable structural features for estimating the extrinsic parameters.

### 4.1. Image-Plane Representation and Boundary Mask Extraction

**Figure 3:**
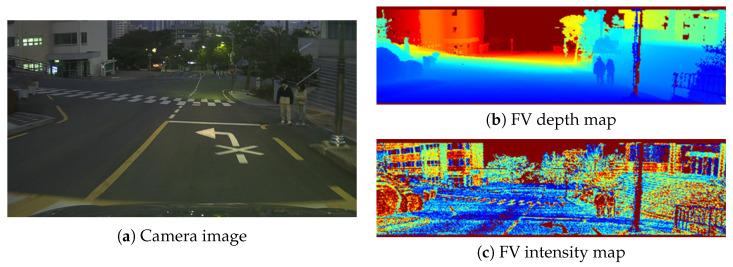
Camera image and corresponding FV LiDAR maps. The FV depth map is color-coded according to depth values using a colormap, while the FV intensity map is color-coded based on normalized intensity values.

This subsection describes the input construction pipeline used by the subsequent PAIRS-Calib procedure. As illustrated in Figure 3, LiDAR pointclouds are projected into the camera viewing domain to form FV representations that share the same image-plane coordinates as the RGB image. The camera resolution defines the pixel grid, while the LiDAR configuration determines the effective sampling density within this grid. By restricting the LiDAR’s full $360^{\circ }$ field of view to the camera’s effective viewing angle, both modalities are aligned within a common spatial domain, enabling direct comparison in the image plane. The resulting FV depth map encodes range information per pixel, and the FV intensity map encodes reflectance per pixel, as shown in Figure 3b and Figure 3c, respectively.

To improve the stability of intensity-based boundary extraction, LiDAR intensity is normalized to reduce depth-dependent attenuation. Specifically, depth values are discretized into bins with interval Δd, and each measurement is normalized using the average intensity of its depth bin. This produces a depth-invariant intensity representation that remains comparable across different ranges, which is beneficial for boundary detection.

**Figure 4:**
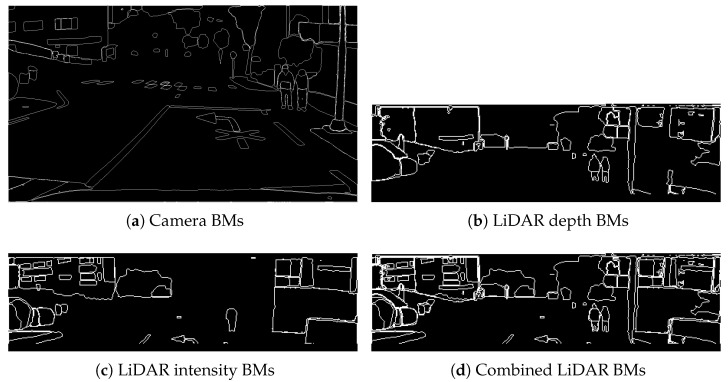
Class-agnostic BMs extracted from (**a**) camera image, (**b**) LiDAR depth map, and (**c**) LiDAR intensity map, with (**d**) their fusion into a combined LiDAR BM.

Building upon these image-plane depth and intensity representations, BM detection are extracted as compact structural inputs for PAIRS-Calib. For each input image (camera RGB, FV depth, and FV intensity), *S* seed points are sampled uniformly over the image grid and fed into a class-agnostic segmentation model to generate local region masks. These masks are produced as SAM-style class-agnostic segmentation outputs. From the resulting segmentation masks, boundary pixels are mapped to 1 while both interior and exterior regions are set to 0, yielding a binary BM as illustrated in Figure 4. The resulting camera BM and the LiDAR depth/intensity BMs are finally used as the modality-aligned structural inputs to PAIRS-Calib, as introduced in Section 4.2 and used throughout Section 4.3, Section 4.4 and Section 4.5.

### 4.2. Overview of the PAIRS-Calib Procedure

The proposed PAIRS-Calib framework integrates the three processing stages—mask matching, global initialization, and SPSA refinement—into a unified calibration pipeline. Given a set of *F* frames, the optimal extrinsic parameters $\theta^{*}$ in (6) are estimated by jointly evaluating candidate poses constructed from BM correspondences across all frames. The candidates are assessed according to the consistency between LiDAR- and camera-derived BMs, and SPSA refinement is applied to obtain a stable estimate.

To formalize the overall procedure, Algorithm 1 presents a high-level overview of PAIRS-Calib, which integrates mask-level association, global initialization, and SPSA-based refinement into a unified calibration pipeline. The inputs consist of LiDAR pointclouds ${\left\{L_{f}\right\}}_{f=1}^{F}$ and camera images ${\left\{I_{f}\right\}}_{f=1}^{F}$ over *F* frames, together with camera intrinsics $K_{int}$, an initial extrinsic estimate $\theta_{\mathrm{init}}$, and a bounded 6-DoF search domain B centered around this initial pose. In addition, the algorithm takes hyperparameters controlling global sampling and refinement, including the number of global samples $N_{\mathrm{glob}}$, the number of retained candidates $K_{\mathrm{top}}$, and the maximum number of SPSA iterations $K_{\max}$. For clarity, we denote by $B_{R}$ the rotational subspace of B used for rotation-only sampling in global initialization, while the translation components are fixed to those of $\theta_{\mathrm{init}}$. The overall procedure is organized into three conceptual stages.
**Algorithm 1** PAIRS-Calib: mask matching, global initialization, and SPSA refinement**Input:** LiDAR pointclouds ${\left\{L_{f}\right\}}_{f=1}^{F}$, Camera images ${\left\{I_{f}\right\}}_{f=1}^{F}$, Intrinsic matrix $K_{int}$, Initial pose $\theta_{\mathrm{init}}$, Search bounds B, Number of global samples $N_{\mathrm{glob}}$, Number of top candidates $K_{\mathrm{top}}$, Maximum SPSA iterations $K_{\max}$**Output:** Sequence-level extrinsic estimate $\theta^{★}$                                            ▹ *Stage 1: Mask Matching (ROI clipping and shape-aware pairing)*  1:**for** f=1…F **do**  2:    Project LiDAR points $L_{f}$ to image plane under $\theta_{\mathrm{init}}$  3:    Extract projected LiDAR masks {r} and image masks {s} from $I_{f}$  4:    **for** each (r,s) **do**  5:        Compute pairwise score matrix ${\mathrm{Score}}_{f}\left(r,s\right)$                             ▹ *As defined in (12)*  6:    **end for**  7:    $M_{f}\leftarrow$ HungarianAssignment $\left({\mathrm{Score}}_{f}\left(r,s\right)\right)$  8:**end for**                                                       ▹ *Stage 2: Global Initialization (rotation-only sampling)*  9:Generate rotational samples ${\left\{\Delta \phi^{\left(n\right)},\Delta \theta^{\left(n\right)},\Delta \psi^{\left(n\right)}\right\}}_{n=1}^{N_{\mathrm{glob}}}∼\mathrm{Uniform}\left(B_{R}\right)$10:**for** $n=1\ldots N_{\mathrm{glob}}$**do**11:    Form 6-DoF pose $\theta^{\left(n\right)}$ by combining sampled rotation with translation of $\theta_{\mathrm{init}}$12:    $L^{\left(n\right)}\leftarrow L\left(\theta^{\left(n\right)};{\left\{M_{f}\right\}}_{f=1}^{F}\right)$                                                                     ▹ *As defined in (18)*13:**end for**14:Select top candidates with the smallest $L^{\left(n\right)}$ ${\left\{\theta_{0}^{\left(m\right)}\right\}}_{m=1}^{K_{\mathrm{top}}}\leftarrow {\mathrm{TopK}}_{\min}\left(\left\{\theta^{\left(n\right)},L^{\left(n\right)}\right\}\right)$                                              ▹ *Stage 3: SPSA Refinement and Joint Integration over (m,f,j)*15:Construct the joint index set $J=\{\left(m,f,j\right)|m=1\ldots K_{\mathrm{top}},\;f=1\ldots F,\;j=1\ldots J_{f}\}$16:**for each** (m,f,j)∈J **do**17:    Initialize $\theta_{m,f,j}\leftarrow \theta_{0}^{\left(m\right)}$18:    Refine $\theta_{m,f,j}$ by SPSA for $K_{\max}$ steps within B:19:    Draw $\Delta_{k}\in {\left\{-1,+1\right\}}^{6}$; Evaluate $L(\theta_{m,f,j}\pm c_{k}\Delta_{k})$;20:    Estimate ${\hat{g}}_{k,m,f,j}$ from the two losses; Update $\theta_{m,f,j}\leftarrow \mathrm{Clamp}\left(\theta_{m,f,j}-\alpha_{k}{\hat{g}}_{k,m,f,j},B\right)$21:    Store $\theta_{m,f,j}^{★}\leftarrow \theta_{m,f,j}$ and $L_{m,f,j}^{★}\leftarrow L\left(\theta_{m,f,j}^{★}\right)$22:**end for**                                                                                             ▹ *Joint gating and aggregation*23:$I\leftarrow \{\left(m,f,j\right)|L_{m,f,j}^{★}\leq \tau_{\mathrm{gate}}\}$24:$w_{m,f,j}\leftarrow \sqrt{\left|P_{f,r_{j}}^{3D}\right|}\;\cdot \;{\left[\max\left(L_{m,f,j}^{★},\epsilon \right)\right]}^{4}\;\forall \left(m,f,j\right)\in I$                            ▹ *As defined in (24)*25:$\theta^{★}\leftarrow \left(\sum_{(m,f,j)\in I}w_{m,f,j}\;\theta_{m,f,j}^{★}\right)/\left(\sum_{(m,f,j)\in I}w_{m,f,j}\right)$                                    ▹ *As defined in (25)*26:**return** $\theta^{★}$

The first stage performs mask matching at the frame level. For each frame *f*, LiDAR points are projected onto the image plane under the initial pose $\theta_{\mathrm{init}}$. Since the LiDAR horizontal FoV is defined with sufficient margin, only the projected points that fall within the image frame are retained for subsequent processing, while the image domain itself remains fixed. Two-dimensional masks are then extractedfrom the camera image, and candidate 2D–2D matching scores are computed between projected LiDAR masks and camera masks based on overlap (IoU), coverage, and shape consistency. The optimal one-to-one correspondences are then obtained by maximizing the total matching score via Hungarian assignment, yielding a frame-wise mapping set $M_{f}$. These correspondences form the observation model used in the global initialization and refinement stages.

The second stage carries out global initialization. Instead of directly optimizing all six DoF, PAIRS-Calib first samples $N_{\mathrm{glob}}$ candidate poses from the rotational subspace $B_{R}$, while keeping the translational components fixed to those of the initial pose $\theta_{\mathrm{init}}$. Each sampled pose $\theta^{\left(n\right)}$ is evaluated using a pose-dependent misalignment loss $L^{\left(n\right)}$ in (18), which aggregates the frame-wise boundary alignment cost across all frames based on the matched pairs ${\left\{M_{f}\right\}}_{f=1}^{F}$. The $K_{\mathrm{top}}$ candidates with the smallest loss values are retained as promising initializations for subsequent SPSA refinement.

The third stage performs SPSA-based refinement on the $K_{\mathrm{top}}$ retained candidates [19]. For each candidate index *m*, the SPSA updates are applied to the 6-DoF parameter vector $\theta_{0}^{\left(m\right)}$ within the bounded domain B for $K_{\max}$ iterations. At iteration *k*, a Rademacher perturbation vector $\Delta_{k}\in {\left\{-1,+1\right\}}^{6}$ is sampled to form two perturbed poses, which are evaluated using the same pose-dependent misalignment loss $L^{\left(n\right)}$ in (18). A stochastic gradient estimate is computed from the difference of the two loss values, and the parameters are updated with step size $\alpha_{k}$ while enforcing the bounds via Clamp(·,B). Here, $\alpha_{k}$ and the SPSA perturbation gain $c_{k}$ are refinement hyperparameters. A small positive constant ϵ is introduced as a loss floor to ensure numerical stability in the confidence weighting and normalization steps, and $|P_{f,r_{j}}^{3D}|$ denotes the cardinality (i.e., the number of 3D LiDAR points) of the matched subset associated with mask $r_{j}$ at frame *f*, which serves as a pair-wise confidence factor in the aggregation weight definition. All these settings, including the loss-based gating threshold $\tau_{\mathrm{gate}}$, will be clarified in Section 4.5. Repeating this procedure over the joint index (m,f,j) yields refined solutions $\theta_{m,f,j}^{★}$ and their losses $L_{m,f,j}^{★}$. The final extrinsic estimate $\theta^{★}$ is then obtained by pooling all refined solutions, applying a loss-based gating threshold, and computing a confidence-weighted average of the retained poses.

Together, these three stages define the core design of PAIRS-Calib: frame-wise mask association establishes reliable cross-modal correspondences; global rotational sampling improves robustness against poor local minima through exploration of the bounded search space; and SPSA refinement enables efficient gradient-free optimization of the extrinsic parameters. The following subsections detail the matching metrics, loss formulation, and optimization strategy.

### 4.3. Mask Matching via IoU–Coverage–Shape Screening

This subsection constructs reliable 2D–2D mask correspondences that remain fixed throughout the global initialization and SPSA refinement stages. The goal is to obtain a strict one-to-one mapping set $M_{f}$ for each frame *f*, where each pair links a LiDAR mask label $r\in S_{f}^{3D}$ to a camera mask label $s\in S_{f}^{2D}$. Since ${\left\{M_{f}\right\}}_{f=1}^{F}$ is used in the computation of $L^{\left(n\right)}$ in Algorithm 1, this stage suppresses ambiguous or spurious associations that could otherwise destabilize the downstream optimization, by enforcing geometric consistency at the bounding-box level and structural consistency at the pixel level.

Given a camera segmentation map $S_{f}^{2D}$, a binary mask $M_{f,s}^{2D}$ is formed for each $s\in S_{f}^{2D}$. On the LiDAR side, we reuse the 3D points associated with the LiDAR masks constructed in Section 4.1. Specifically, for each $r\in S_{f}^{3D}$, the corresponding point subset is denoted by $P_{f,r}^{3D}$. Using the initial pose $\theta_{\mathrm{init}}$, $P_{f,r}^{3D}$ is projected into the image plane to form a binary projection mask $M_{f,r}^{3D}$. For geometric consistency evaluation, bounding boxes $B_{f,s}$ and $B_{f,r}$ are computed from $M_{f,s}^{2D}$ and $M_{f,r}^{3D}$, respectively, and their areas are used in the subsequent overlap and coverage definitions.

Candidate associations (r,s) are evaluated by measuring overlap consistency at the bounding-box level. Let $I_{f}\left(r,s\right)$ and $U_{f}\left(r,s\right)$ denote the intersection and union of the two masks, respectively. The intersection-over-union is defined as

(7) $${\mathrm{IoU}}_{f}\left(r,s\right)=\frac{I_{f}\left(r,s\right)}{U_{f}\left(r,s\right)}=\frac{|B_{f,r}\cap B_{f,s}|}{|B_{f,r}\cup B_{f,s}|}.$$

In addition, two directional coverage measures are computed based on the bounding-box areas as

(8) $${\mathrm{Cov}}^{2D}\left(r,s\right)=\frac{I_{f}\left(r,s\right)}{|B_{f,s}|},\;{\mathrm{Cov}}^{3D}f\left(r,s\right)=\frac{I_{f}\left(r,s\right)}{|B_{f,r}|},$$

and combined into a symmetric fused coverage term

(9) $${\mathrm{CovF}}_{f}\left(r,s\right)=\frac{2\;{\mathrm{Cov}}_{f}^{2D}\left(r,s\right)\;{\mathrm{Cov}}_{f}^{3D}\left(r,s\right)}{{\mathrm{Cov}}_{f}^{2D}\left(r,s\right)+{\mathrm{Cov}}_{f}^{3D}\left(r,s\right)}.$$

To improve the stability of the fixed correspondence set $M_{f}$ under noisy or fragmented class-agnostic masks, a shape-consistency term is introduced. Unlike the overlap and coverage terms, which are computed at the bounding-box level, the shape-consistency term operates directly on pixel and projected point coordinates to preserve fine-grained structural alignment. Principal component analysis is applied to the pixel coordinates of $M_{f,s}^{2D}$ and to the image-plane coordinates of the projected 3D points $\Pi (P_{f,r}^{3D};\theta_{\mathrm{init}})$. Let $\lambda_{1}^{2D}\geq \lambda_{2}^{2D}>0$ and $\lambda_{1}^{3D}\geq \lambda_{2}^{3D}>0$ denote the eigenvalues of the respective covariance matrices. The corresponding aspect ratios are defined as

(10) $${\mathrm{AR}}_{f,s}^{2D}=\sqrt{\frac{\lambda_{1}^{2D}}{\lambda_{2}^{2D}}},\;{\mathrm{AR}}_{f,r}^{3D}=\sqrt{\frac{\lambda_{1}^{3D}}{\lambda_{2}^{3D}}}.$$

The shape-consistency score is then computed as

(11) $$Shape_{f}\left(r,s\right)=\exp\;\left(-\left|\log\;\left(\frac{{\mathrm{AR}}_{f,r}^{3D}}{{\mathrm{AR}}_{f,s}^{2D}}\right)\right|\right),$$

which approaches 1 when the projected 3D structure and the 2D mask exhibit similar elongation and decreases as their aspect ratios diverge.

The final candidate score used for association is defined as

(12) $${\mathrm{Score}}_{f}\left(r,s\right)=w_{\mathrm{IoU}}{\mathrm{IoU}}_{f}\left(r,s\right)+w_{\mathrm{Cov}}{\mathrm{CovF}}_{f}\left(r,s\right)+w_{\mathrm{Shape}}Shape_{f}\left(r,s\right),$$

subject to hard gates on ${\mathrm{IoU}}_{f}\left(r,s\right)\;\geq \;\tau_{\mathrm{IoU}}$, ${\mathrm{Cov}}_{f}^{2D}\left(r,s\right)\;\geq \;\tau_{C2D}$, and ${\mathrm{Cov}}_{f}^{3D}\left(r,s\right)\;\geq \;\tau_{C3D}$. The score ${\mathrm{Score}}_{f}\left(r,s\right)$ is used for ranking and assignment, while the hard gates remove pairs that lack sufficient geometric overlap support.

Finally, strict one-to-one matching is enforced by formulating the candidate association problem as a bipartite assignment and solving it using the Hungarian algorithm [20]. All candidate pairs that satisfy the gating conditions are collected, and a cost matrix is constructed from the negative matching scores $-{\mathrm{Score}}_{f}\left(r,s\right)$, such that maximizing the total matching score is equivalent to minimizing the assignment cost. The Hungarian algorithm yields a globally optimal one-to-one correspondence, producing the mapping set

(13) $$M_{f}={\left(r_{j},s_{j}\right)}_{j=1}^{J_{f}},$$

which is free from one-to-many or many-to-one ambiguities by construction. Each matching $\left(r_{j},s_{j}\right)$ stores the corresponding projected LiDAR mask $M_{f,r_{j}}^{3D}$ and the 2D image mask $M_{f,s_{j}}^{2D}$, along with their overlap statistics. These fixed correspondences provide the observation model consumed by $L^{\left(n\right)}$ in Algorithm 1.

### 4.4. Global Initialization and Pose-Dependent Misalignment Loss

This subsection describes the global initialization stage in Algorithm 1 (Stage 2), which identifies promising extrinsic candidates prior to SPSA refinement. Rather than directly optimizing all six DoF, PAIRS-Calib samples $N_{\mathrm{glob}}$ candidate poses within the bounded search space B. More specifically, sampling focuses on the rotational components $\left(\Delta \phi ,\Delta \theta ,\Delta \psi \right)∼\mathrm{Uniform}\left(B_{R}\right)$, while the translational components are fixed to those of the initial pose $\theta_{\mathrm{init}}$, yielding the DoF candidates ${\left\{\theta^{\left(n\right)}\right\}}_{n=1}^{N_{\mathrm{glob}}}$.

Following Algorithm 1, each candidate is evaluated by a misalignment loss $L^{\left(n\right)}$ and the top-$K_{\mathrm{top}}$ candidates ${\left\{\theta_{0}^{\left(m\right)}\right\}}_{m=1}^{K_{\mathrm{top}}}$ are selected via ${\mathrm{TopK}}_{\min}\left(\left\{\theta^{\left(n\right)},L^{\left(n\right)}\right\}\right)$ and forwarded to the SPSA stage (Stage 3). Importantly, $L^{\left(n\right)}$ is a pose-dependent geometric misalignment loss that is distinct from the association score used to construct $M_{f}$ in Section 4.3.

To compute $L^{\left(n\right)}$, PAIRS-Calib reuses the fixed correspondences ${\left\{M_{f}\right\}}_{f=1}^{F}$ and evaluates a per-pair loss for each matched pair $\left(r_{j},s_{j}\right)\in M_{f}$ under a candidate pose θ. For a given frame *f* and pair index *j*, projection consistency is evaluated using a distance transform map computed from the camera-derived BM: letting $d_{f,j,i}$ denote the pixel-wise distance between the projected location of the *i*-th valid 3D point and the nearest boundary pixel of the corresponding 2D mask, the point-wise proximity loss is computed as

(14) $$L_{f,j}^{\mathrm{dist}}\left(\theta \right)=1-\frac{1}{N_{f,j}}\sum_{i=1}^{N_{f,j}}\exp\left(-\beta d_{f,j,i}\right),$$

where $N_{f,j}$ is the number of valid projected points for the (f,j) pair, and β controls the spatial sensitivity.

Complementing the distance term, a bounding-box alignment loss is computed between the stored 2D mask box $B_{f,s_{j}}$ and the projected point box $B_{f,r_{j}}^{\mathrm{proj}}\left(\theta \right)$:

(15) $$L_{f,j}^{\mathrm{bbox}}\left(\theta \right)=1-\mathrm{IoU}\;\left(B_{f,s_{j}},\;B_{f,r_{j}}^{\mathrm{proj}}\left(\theta \right)\right).$$

To handle invalid or out-of-image projections, we define the out-of-image ratio for the (f,j) pair as

(16) $$L_{\mathrm{out}}^{f,j}\left(\theta \right)=\frac{N_{\mathrm{inv}}^{f,j}\left(\theta \right)+N_{\mathrm{oob}}^{f,j}\left(\theta \right)}{|P_{f,r_{j}}^{3D}|},$$

where $N_{\mathrm{inv}}^{f,j}\left(\theta \right)$ counts points violating the depth gate ($Z_{c}\leq z_{\min}$) and $N_{\mathrm{oob}}^{f,j}\left(\theta \right)$ counts points projected outside the image bounds.

The final per-pair loss is then defined by linearly combining these three losses

(17) $$L_{f,j}\left(\theta \right)=w_{\mathrm{dist}}L_{f,j}^{\mathrm{dist}}\left(\theta \right)+w_{\mathrm{bbox}}L_{f,j}^{\mathrm{bbox}}\left(\theta \right)+w_{\mathrm{oob}}L_{\mathrm{out}}^{f,j}\left(\theta \right).$$

Since $L_{f,j}\left(\theta \right)$ is defined at the matching-pair level, the pose-dependent misalignment loss is obtained by aggregating scores over all matched pairs and frames. Let $J_{f}=\left|M_{f}\right|$ denote the number of matched pairs at frame *f*. The pose-dependent misalignment loss used in Stage 2 and Stage 3 is defined as

(18) $$L^{\left(n\right)}=L\;\left(\theta^{\left(n\right)};{\left\{M_{f}\right\}}_{f=1}^{F}\right)≜\frac{1}{\sum_{f=1}^{F}J_{f}}\sum_{f=1}^{F}\;\sum_{j=1}^{J_{f}}L_{f,j}\;\left(\theta^{\left(n\right)}\right).$$

Consequently, lower values of $L^{\left(n\right)}$ indicate better geometric alignment and are favored in both the global candidate selection and the subsequent SPSA refinement.

### 4.5. SPSA-Based Refinement of Top-*K* Candidates

The third stage performs SPSA-based refinement on the $K_{\mathrm{top}}$ candidate poses retained from the global initialization stage. While Stage 2 provides coarse exploration of the bounded search space, this stage focuses on local optimization of the full 6-DoF parameter vector θ with respect to the misalignment loss $L\left(\theta ;{\left\{M_{f}\right\}}_{f=1}^{F}\right)$ in (18).

Importantly, Stage 3 can be executed independently over the joint index (m,f,j), where $m=1,\ldots ,K_{\mathrm{top}}$ denotes the candidate index, f=1,…,F the frame index, and $j=1,\ldots ,J_{f}$ the matched pair index. SPSA refinement yields optimized 6-DoF solutions $\theta_{m,f,j}^{★}$ together with their corresponding loss values $L_{m,f,j}^{★}$. The final extrinsic estimate is obtained by pooling all refined solutions across (m,f,j) and applying a single loss-based gating and confidence-weighted aggregation scheme.

For completeness, the SPSA update rule is summarized below: the parameter vector is iteratively updated for $K_{\max}$ steps, and at iteration *k* a Rademacher perturbation vector $\Delta_{k}\in {\left\{-1,+1\right\}}^{6}$ is sampled to construct two perturbed candidates.

(19) $$\theta_{m,f,j}^{+}=\mathrm{Clamp}\;\left(\theta_{m,f,j}+c_{k}\Delta_{k},B\right),\;\theta_{m,f,j}^{-}=\mathrm{Clamp}\;\left(\theta_{m,f,j}-c_{k}\Delta_{k},B\right),$$

where $\theta_{m,f,j}$ denotes the current iterate associated with the joint index (m,f,j), $c_{k}$ controls the perturbation magnitude, and Clamp(·,B) enforces the bounded search domain. The stochastic gradient estimate for the (m,f,j)-th refinement mask is obtained directly from the pose-dependent misalignment loss as

(20) $${\hat{g}}_{k,m,f,j}=\frac{L\;\left(\theta_{m,f,j}^{+}\right)-L\;\left(\theta_{m,f,j}^{-}\right)}{2c_{k}}\;\Delta_{k}^{-1},$$

and the parameter vector is updated according to

(21) $$\theta_{m,f,j}\leftarrow \mathrm{Clamp}\;\left(\theta_{m,f,j}-\alpha_{k}{\hat{g}}_{k,m,f,j},B\right),$$

where $\alpha_{k}$ denotes the step size. After $K_{\max}$ iterations, SPSA refinement under the *m*-th initialization yields pair-wise optimized poses $\theta_{m,f,j}^{★}$ and their corresponding loss values

(22) $$L_{m,f,j}^{★}≜L\;\left(\theta_{m,f,j}^{★};{\left\{M_{f}\right\}}_{f=1}^{F}\right).$$

To obtain the final extrinsic estimate, we collect all refined solutions across candidates and pairs, apply a single loss-based gating rule,

(23) $$I=\{\left(m,f,j\right)|L_{m,f,j}^{★}\leq \tau_{\mathrm{gate}}\},$$

and assign confidence weights to the retained 3-tuples as

(24) $$w_{m,f,j}=\sqrt{|P_{f,r_{j}}^{3D}|}\;\cdot \;{\left[\max(L_{m,f,j}^{★},\epsilon )\right]}^{4},$$

where loss floor ϵ prevents numerical instability. The final 6-DoF extrinsic estimate is then obtained by a single confidence-weighted aggregation over I,

(25) $$\theta^{★}=\frac{\sum_{(m,f,j)\in I}w_{m,f,j}\;\theta_{m,f,j}^{★}}{\sum_{(m,f,j)\in I}w_{m,f,j}}.$$

By pooling refined solutions across the joint index (m,f,j), this formulation retains the robustness benefits of multiple initializations while avoiding redundant two-stage aggregation, thereby yielding a concise and consistent integration rule.

## 5. Experimental Results

This section presents the experimental assessment of the proposed PAIRS-Calib framework. Section 5.1 describes the experimental setup and evaluation protocols for both the KITTI dataset [21] and our in-house vehicle platform. Section 5.2 reports KITTI benchmark results, directly comparing rotational and translational errors, as well as runtime, against CalibAnything [10]. Finally, Section 5.3 presents real driving results on the in-house platform to demonstrate robustness to synchronization disturbances and pose variations.

### 5.1. Experimental Setup

This section evaluates the proposed PAIRS-Calib on both a public benchmark dataset and a real-world vehicle platform to ensure reproducibility and practical validation. For benchmark evaluation, we use the KITTI dataset [21], which provides synchronized LiDAR–camera pairs with ground-truth poses. The KITTI setup includes a 64-channel Velodyne LiDAR (Velodyne Lidar Inc., San Jose, CA, USA) and a forward-facing monocular camera, enabling direct computation of rotational and translational errors with respect to the ground-truth extrinsic parameters. Section 5.2 reports the KITTI benchmark comparison against CalibAnything [10] using rotational error, translational error, and runtime.

**Figure 5:**
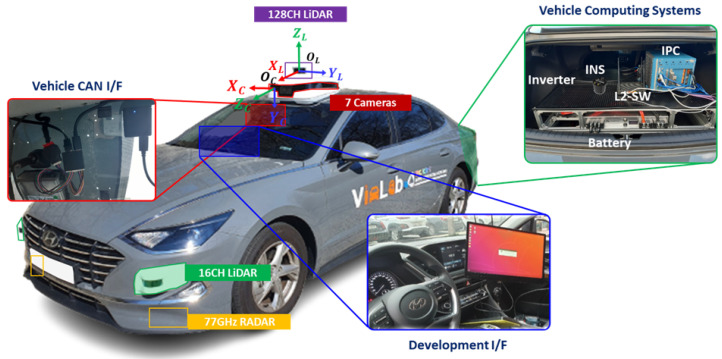
Transformation chain from LiDAR coordinate to image coordinate for extrinsic calibration.

Figure 5 shows the experimental vehicle platform, based on a Hyundai SONATA Dn8, which integrates multiple subsystems to support autonomous driving research. A roof-mounted Ouster OS-1-128 LiDAR (Ouster Inc., San Francisco, CA, USA) serves as the primary ranging sensor. Directly beneath the LiDAR, six short-range rolling-shutter Leopard LI-USB30-M021XB cameras (Leopard Imaging Inc., Fremont, CA, USA) are installed at 60° intervals to provide panoramic coverage, and a single long-range camera is co-located for forward perception. In this study, extrinsic calibration was performed between the 128-channel LiDAR and the forward-facing short-range camera, as indicated in Figure 5. A 16-channel LiDAR and a 77 GHz radar are also integrated on the platform but are not used in this work. The sensors were synchronized using recorded timestamps; in our system, the forward-facing camera is triggered via a ROS topic when the LiDAR scan reaches the camera-facing direction. As a result, residual triggering latency and timestamp uncertainty may occur, leading to slight temporal misalignment between the LiDAR and camera measurements.

The experimental parameters are summarized in Table 2. These include general settings such as the number of frames, segmentation seeds, and the rotational/translational search ranges used for global initialization and SPSA-based refinement. Sensor-specific parameters for both KITTI and the in-house platform are also provided (e.g., camera resolution/FoV and LiDAR channel count/frame rate), together with mask-matching gates and score weights, pose-dependent misalignment loss parameters (spatial sensitivity, loss weights, and depth gate), and optimization hyperparameters to ensure reproducibility across both datasets.

Outdoor experiments were conducted on the Pusan National University campus along a 50 s driving route, which included lane markings, sidewalks, vegetation, and pedestrian crossings. Because the objective is to examine robustness under practical driving conditions, the calibration is performed in a frame-wise manner by setting the number of frames to F=1 in PAIRS-Calib. This configuration avoids implicit temporal smoothing and allows each frame to be evaluated independently. Due to inter-sensor time offsets and rolling-shutter effects, small pose disturbances may arise between LiDAR and camera measurements during motion. Since such dynamic disturbances prevent the definition of reliable per-frame ground truth, the outdoor evaluation is designed to assess qualitative alignment behavior rather than absolute error metrics.

### 5.2. Quantitative Evaluation on the KITTI Dataset

This section quantitatively evaluates the proposed PAIRS-Calib framework on the KITTI dataset [21], assessing estimation accuracy and computational efficiency using rotational error (deg), translational error (m), and average runtime (s). To examine how performance varies with the amount of geometric information, the number of frames was varied over F∈{1,2,4,8,16,32}. For each setting, 100 initial extrinsic poses were sampled from a uniform distribution within the bounded search space defined in Table 2, centered at the KITTI ground-truth extrinsic parameters. Each algorithm was then executed independently from these randomized initializations, and the reported results correspond to averages over the 100 trials, thereby reducing bias from favorable starting conditions.

**Figure 6:**
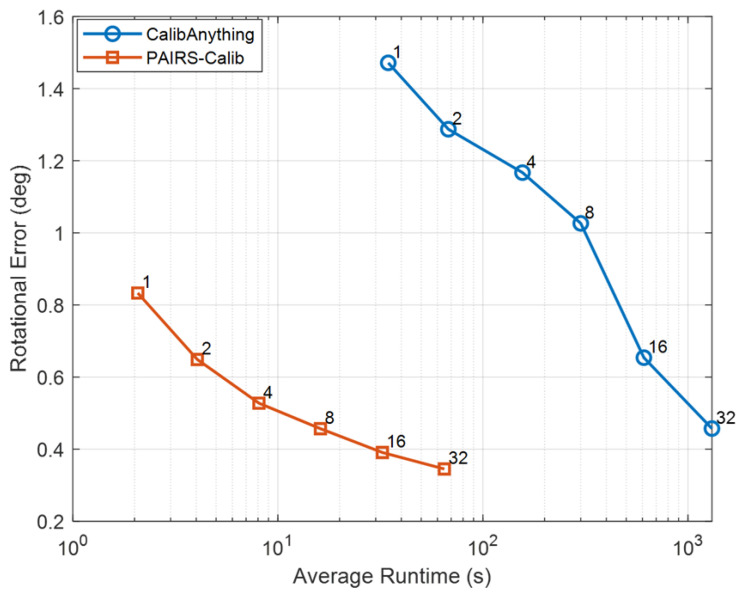
Pareto analysis on KITTI: rotational error versus average runtime. Each marker is annotated with the number of frames *F*.

Figure 6 shows the Pareto relationship between runtime and rotational error, highlighting the fundamental scaling difference between PAIRS-Calib and CalibAnything. As the number of frames increases, CalibAnything gradually reduces rotational error from 1.287° at F=1 to 0.457° at F=32; however, this improvement is accompanied by a substantial increase in runtime from 34.54 s to 1308.94 s. In contrast, PAIRS-Calib consistently achieves lower rotational error across all frame counts while maintaining substantially reduced runtime. For instance, at F=32, PAIRS-Calib attains 0.345° within only 64.86 s, and even at F=1 it already yields significantly smaller rotational error (0.833° vs. 1.287°) with more than an order-of-magnitude reduction in runtime. Considering that rotational error is the dominant factor influencing projection consistency and overall extrinsic calibration quality, this consistent advantage directly translates into more stable cross-modal alignment. These results therefore demonstrate that PAIRS-Calib forms a consistently superior Pareto frontier in the rotation–runtime space, simultaneously improving geometric accuracy and computational efficiency.

**Figure 7:**
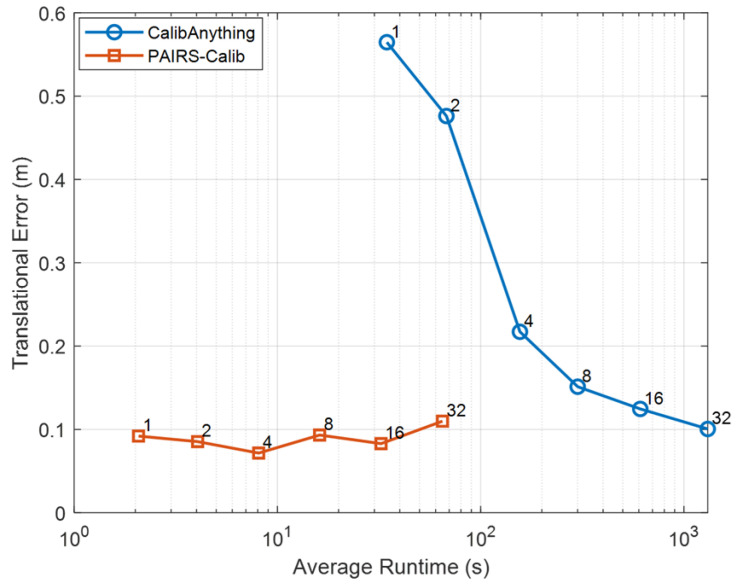
Pareto analysis on KITTI: translational error versus average runtime.

A similar trend is observed in Figure 7, which illustrates translational error versus runtime. While CalibAnything achieves slightly lower translational error at the largest frame count (F=32), reaching 0.101 m compared to 0.110 m for PAIRS-Calib, the proposed scheme outperforms CalibAnything across most practical operating regimes with small-to-moderate frame counts. In particular, for F≤16, PAIRS-Calib consistently attains lower translational error while maintaining significantly lower runtime. Notably, even in the single-frame configuration (F=1), PAIRS-Calib achieves 0.092 m translational error with 2.07 s runtime, whereas CalibAnything reports 0.565 m with 34.54 s. These results indicate that the proposed global initialization and SPSA refinement scheme effectively recovers extrinsic parameters without relying on extensive temporal aggregation, while preserving competitive accuracy at larger frame counts.

**Figure 8:**
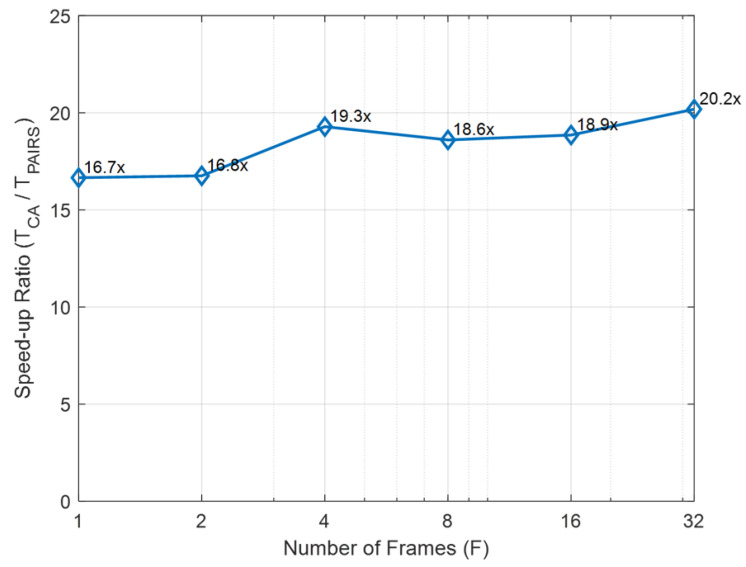
Runtime speed-up ratio defined as $T_{\mathrm{CA}}/T_{\mathrm{PAIRS}}$ across different frame counts.

The computational advantage is further quantified in Figure 8, which reports the runtime speed-up ratio $T_{\mathrm{CA}}/T_{\mathrm{PAIRS}}$ across frame counts. The speed-up remains consistently between approximately 16 and 20 times, indicating stable efficiency gains as *F* increases. This improvement results from the balanced combination of three efficiency-oriented components in Section 4.3, Section 4.4 and Section 4.5. First, the mask matching in Section 4.3 suppresses unreliable class-agnostic segmentation outputs through IoU–coverage–shape screening and one-to-one assignment, ensuring that subsequent optimization focuses on a compact set of reliable correspondences. Second, the global initialization strategy in Section 4.4 efficiently identifies promising starting poses via bounded rotation-only sampling, which reduces unnecessary exploration of the full 6-DoF space while remaining highly sensitive to boundary misalignment. Third, the SPSA refinement in Section 4.5 further lowers computational burden while preserving convergence properties reported in prior work [19], since it avoids full-gradient computation and instead updates parameters using a stochastic estimate constructed from only two loss evaluations per iteration. Importantly, these runtime savings are not achieved by sacrificing accuracy; rather, PAIRS-Calib maintains competitive (often better) calibration performance while forming a consistently superior accuracy–efficiency trade-off across operating points.

Overall, the KITTI benchmark demonstrates that PAIRS-Calib delivers a substantially improved accuracy–efficiency trade-off, supporting both frame-wise calibration and scalable multi-frame refinement with significantly reduced computational burden.

### 5.3. Qualitative Results on the In-House Driving Platform (F = 1)

This section presents qualitative results of PAIRS-Calib obtained from real driving experiments on the in-house vehicle platform under the frame-wise setting (F=1). During on-road driving, inter-sensor time offsets and rolling-shutter effects can induce small but non-negligible pose disturbances between the LiDAR scan and the camera image, which may appear as frame-to-frame fluctuations in the effective extrinsic parameters. Because such dynamic disturbances prevent the definition of a reliable per-frame ground truth extrinsic, the performance is assessed visually by examining cross-modal boundary alignment in representative scenes, rather than by absolute error metrics or target-based references.

**Figure 9:**
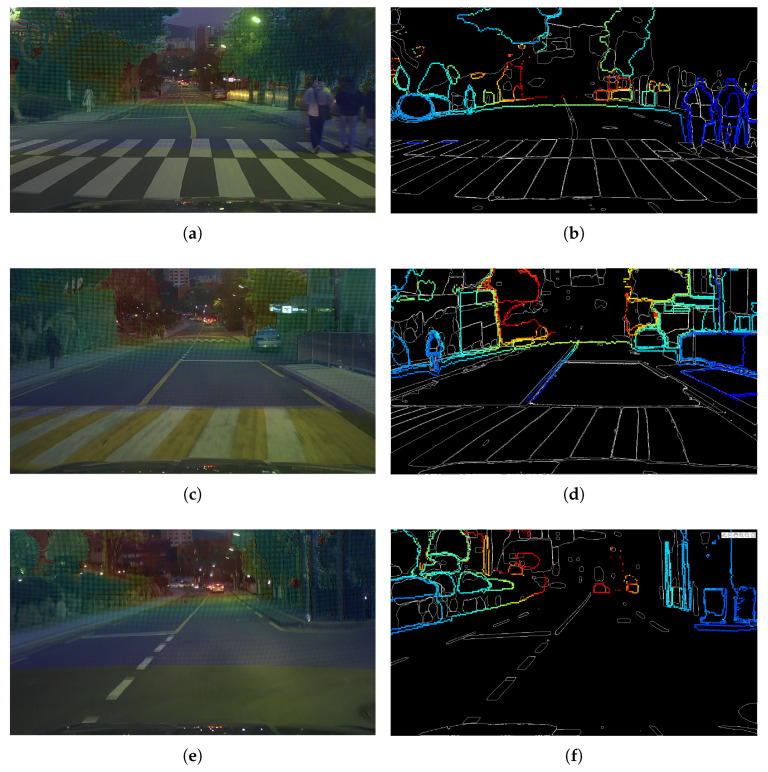
Qualitative frame-wise (F=1) results of PAIRS-Calib on the in-house driving platform. Depth overlays (**left**) and projected LiDAR boundary/edge overlays (**right**) illustrate consistent cross-modal alignment under practical driving conditions, where inter-sensor time offsets and rolling-shutter effects may introduce frame-level pose disturbances. In all overlays, the LiDAR pointcloud is color-coded according to depth values using a colormap. (**a**) Depth overlay over camera image 1. (**b**) Projected LiDAR edges over camera image 1. (**c**) Depth overlay over camera image 2. (**d**) Projected LiDAR edges over camera image 2. (**e**) Depth overlay over camera image 3. (**f**) Projected LiDAR edges over camera image 3.

To qualitatively assess calibration performance, Figure 9 shows representative samples where the projected LiDAR structures are overlaid on the camera image after frame-wise extrinsic recovery. Each sample is organized into two panels: Figure 9a,c,e show depth overlays on the camera images to visualize range-consistent projection behavior, whereas Figure 9b,d,f show projected LiDAR boundary overlays to highlight boundary-level alignment with salient scene structures (e.g., lane markings, sidewalks, vegetation, and pedestrians). Across the three examples, the overlays remain geometrically consistent despite potential synchronization-induced pose variations, indicating that PAIRS-Calib can restore stable extrinsic alignment under practical driving disturbances.

## 6. Conclusions

This paper presented *PAIRS-Calib*, a targetless LiDAR–camera extrinsic calibration framework that estimates 6-DoF extrinsics using class-agnostic boundary masks in a common image-plane representation. PAIRS-Calib constructs reliable cross-modal mask pairs using overlap/coverage and lightweight shape-consistency cues, computes cost-efficient coarse initialization candidates through bounded rotation-only exploration, and refines candidates via SPSA using a pose-dependent geometric misalignment loss defined on image-plane boundary and region consistency. By integrating multiple pairwise pose hypotheses rather than relying on a single optimization trajectory, the proposed pipeline improves robustness to practical disturbances such as segmentation noise, vehicle vibration, and inter-modal timing jitter, while maintaining a favorable accuracy–runtime balance on both a public benchmark and a real driving platform. Future work will focus on improving real-time capability by accelerating class-agnostic mask extraction and pair construction, reducing candidate evaluations through adaptive sampling and warm-start strategies, and exploiting temporal continuity for incremental updates. For practical applicability, further validation under challenging conditions, such as severe occlusions, adverse weather, and highly dynamic scenes, will be pursued to strengthen robustness.

## Figures and Tables

**Table 1:** Comparison of representative targetless LiDAR–camera calibration approaches.

Approach	LiDAR Representation	Camera Representation	Matching Space	Inference Paradigm
MFCalib [6]	3D (Multi-feature edges)	Front view (FV) (Canny edges)	3D–2D	Geometric optimization
BEVCalib [9]	Bird’s-Eye-View (BEV) (Voxelization)	BEV (visual features)	2D–2D	Global optimization in BEV space
CalibRefine [7]	FV (2D projection + visual features)	FV (neural network backbone)	2D–2D	Learning-based latent alignment
CalibAnything [10]	Implicit (Attribute-based cues)	FV (class-agnostic masks)	3D–2D	Global search with projection consistency
PAIRS-Calib (Ours)	FV (class-agnostic BMs)	FV (class-agnostic BMs)	2D–2D	Coarse initialization + SPSA refinement

**Table 2:** Parameters of KITTI and vehicle platform experiments.

General Parameters	KITTI	Platform	Unit
Number of Frames (*F*)	{1, 2, 4, 8, 16, 32}	1	-
SAM Seg. Seeds (*S*)	1024	1024	-
Rotational Search Space (Δϕ,Δθ,Δψ)	$\pm 2.5^{\circ }$	$\pm 2.5^{\circ }$	deg
Translational Search Space (Δx,Δy,Δz)	±0.25	±0.25	m
**Camera Parameters**	**KITTI**	**Platform**	**Unit**
Horizontal field of view (FoV)	82	80	°
Vertical FoV	30	45	°
Frames per second (FPS)	10	10	Hz
Resolution	1241 × 376	1280 × 720	pixels
**LiDAR Parameters**	**KITTI**	**Platform**	**Unit**
Vertical Channels	64	128	-
Horizontal Resolution	0.176	0.176	°
FPS	10	10	Hz
Depth-bin Interval (Δd)	-	0.5	m
**Mask Matching Parameters**	**KITTI**	**Platform**	**Unit**
IoU Gate ($\tau_{\mathrm{IoU}}$)	0.10	0.10	-
2D Coverage Gate ($\tau_{C2D}$)	0.05	0.05	-
3D Coverage Gate ($\tau_{C3D}$)	0.25	0.25	-
Score Weights ($w_{\mathrm{IoU}},w_{\mathrm{Cov}},w_{\mathrm{Shape}}$)	(0.3, 0.5, 0.2)	(0.3, 0.5, 0.2)	-
**Sequence-Level Loss Parameters**	**KITTI**	**Platform**	**Unit**
Spatial Sensitivity (β)	0.1	0.1	${\mathrm{pixel}}^{-1}$
Loss Weights ($w_{\mathrm{dist}},w_{\mathrm{bbox}},w_{\mathrm{oob}}$)	(0.7, 0.3, 0.5)	(0.7, 0.3, 0.5)	-
Depth Gate ($z_{\min}$)	0.5	0.5	m
**Global Init and SPSA Parameters**	**KITTI**	**Platform**	**Unit**
Number of Global Samples ($N_{\mathrm{glob}}$)	500	500	-
Top-*K* Pose Selection ($K_{\mathrm{top}}$)	20	20	-
Max SPSA Iterations ($K_{\max}$)	80	80	-
Perturbation Gain ($c_{k}$)	0.001	0.001	-
Step Size ($\alpha_{k}$)	0.005	0.005	-
Gate Threshold ($\tau_{\mathrm{gate}}$)	0.0	0.0	-
Loss Floor (ϵ)	0.05	0.05	-

## Data Availability

The original contributions presented in this study are included in the article. Further inquiries can be directed to the corresponding author.
